# Photon-counting computed tomography of coronary and peripheral artery stents: a phantom study

**DOI:** 10.1038/s41598-023-41854-3

**Published:** 2023-09-08

**Authors:** Thomas Stein, Jana Taron, Niklas Verloh, Michael Doppler, Alexander Rau, Muhammad Taha Hagar, Sebastian Faby, Dimos Baltas, Dirk Westermann, Isabelle Ayx, Stefan O. Schönberg, Konstantin Nikolaou, Christopher L. Schlett, Fabian Bamberg, Jakob Weiss

**Affiliations:** 1 Department of Diagnostic and Interventional Radiology, Medical Center, Faculty of Medicine, University of Freiburg, Freiburg, Germany; 2 Computed Tomography, Siemens Healthcare GmbH, Forchheim, Germany; 3 Division of Medical Physics, Department of Radiation Oncology, Medical Center University of Freiburg, Faculty of Medicine, University of Freiburg, Freiburg, Germany; 4 German Cancer Consortium (DKTK), Partner Site Freiburg, Freiburg, Germany; 5 Department of Cardiology and Angiology, Interdisciplinary Vascular Center Freiburg-Bad Krozingen, Faculty of Medicine, University of Freiburg, Freiburg, Germany; 6 Department of Radiology and Nuclear Medicine, Medical Faculty Mannheim, University Medical Center Mannheim, University of Heidelberg, Mannheim, Germany; 7 Department of Diagnostic and Interventional Radiology, University Hospital Tübingen, Tübingen, Germany

**Keywords:** Anatomy, Cardiology, Health care

## Abstract

Accurate small vessel stent visualization using CT remains challenging. Photon-counting CT (PCD-CT) may help to overcome this issue. We systematically investigate PCD-CT impact on small vessel stent assessment compared to energy-integrating-CT (EID). 12 water-contrast agent filled stents (3.0–8 mm) were scanned with patient-equivalent phantom using clinical PCD-CT and EID-CT. Images were reconstructed using dedicated vascular kernels. Subjective image quality was evaluated by 5 radiologists independently (5-point Likert-scale; 5 = excellent). Objective image quality was evaluated by calculating multi-row intensity profiles including edge rise slope (ERS) and coefficient-of-variation (CV). Highest overall reading scores were found for PCD-CT-Bv56 (3.6[3.3–4.3]). In pairwise comparison, differences were significant for PCD-CT-Bv56 vs. EID-CT-Bv40 (*p* ≤ 0.04), for sharpness and blooming respectively (all *p* < 0.05). Highest diagnostic confidence was found for PCD-CT-Bv56 (*p* ≤ 0.2). ANOVA revealed a significant effect of kernel strength on ERS (*p* < 0.001). CV decreased with stronger PCD-CT kernels, reaching its lowest in PCD-CT-Bv56 and highest in EID-CT reconstruction (*p* ≤ 0.05). We are the first study to verify, by phantom setup adapted to real patient settings, PCD-CT with a sharp vascular kernel provides the most favorable image quality for small vessel stent imaging. PCD-CT may reduce the number of invasive coronary angiograms, however, more studies needed to apply our results in clinical practice.

## Introduction

Cardiovascular disease is the leading cause of death worldwide and accounts for one in every three deaths in the United States^1^. In patients with flow-limiting coronary artery stenosis (> 70% luminal narrowing), current guidelines recommend percutaneous coronary intervention (PCI) with stent implantation to reduce morbidity and mortality^2^. PCI is a common procedure with an estimated number of 600.000 interventions per year in the United States^3^ and numbers are expected to further increase due to demographic changes^4^. While the primary success rate of PCI is high, recurrent symptoms in this population are frequently encountered with the need for reevaluation of patency^5^. Percutaneous coronary angiography is the current method of choice^6^. However, it is invasive and associated with the risk of peri- and postprocedural complications and may require hospitalization^7^. Thus, non-invasive options for diagnosis, treatment planning and follow-up are desirable.

Over the past decades, coronary computed tomography angiography (CCTA) has emerged as a promising non-invasive diagnostic procedure with a growing body of evidence for reliable diagnosis of coronary artery disease, prognostication and patient management^8^. As a result, CCTA was implemented in recent guidelines and is now recommended as the diagnostic modality of choice in patients with low to intermediate pretest likelihood of coronary artery disease. Moreover, the Society of Cardiovascular Computed Tomography also promotes the use of CCTA after coronary artery revascularization^9^. However, known drawbacks are the impaired image quality due to limited spatial resolution and the blooming artifacts caused from metallic stent struts^10^.

One possibility to overcome these limitations is the recently introduced photon-counting detector (PCD) technology^11^. In contrast to the currently used energy-integrating detectors (EID) that detect and generate a signal proportional to the absorbed photons, PCD-CT allows for counting every single photon arriving at the detector and evaluating its energy^12^. This facilitates improved image reconstruction with the potential to significantly reduce blooming artifacts^13^. In addition, the PCD architecture substantially increases the spatial resolution compared to EID-CT systems^14^. However, little is known about the appropriate reconstruction settings for dedicated stent imaging.

Hence, the aim of this study was to systematically investigate the value of PCD-CT for the assessment of small vessel stents using a patient-equivalent phantom and identify the most favorable protocol settings for future clinical implementation.

## Material and methods

### Ethical approval

The study was approved by Institutional Review Board (No. 21-2469 Ethics Committee, University of Freiburg). All participants provided informed written consent. The study was conducted following the Declarations of Helsinki.

### Phantom setup and stents

All stent imaging studies were performed using an in-house developed stent phantom. To mimic a clinically realistic scenario, the phantom was built to reflect the water-equivalent diameter of an average patient. Therefore, a random sample of consecutive 457 patients (189 women, 268 men; age 61.15 ± 12.95; median BMI 27.2; range 17.2–58.8) who underwent clinically indicated cardiac CT were retrospectively analyzed to calculate the water-equivalent patient diameter using a commercially available dose management system (DoseM, Infinitt EU, Frankfurt, Germany). The water-equivalent diameter was defined according to American Association of Physicists in Medicine (AAPM TG220)^15^:

1 $$D_{W} = 2\sqrt {\left[ {\frac{1}{1000}HU\left( {x,y} \right)_{ROI} + 1} \right]\frac{{A_{ROI} }}{\pi }}$$

where D_W_ is the water equivalent diameter, HU(x,y)_ROI_ the mean CT Hounsfield Units (HU) in the region-of-interest (ROI). A_ROI_ is the total surface of the ROI and equals to the sum of the surface of all pixels in the ROI. The ROI may include the air surrounding the patient, where air-voxels with almost no attenuation have little effect on the accuracy of D_W_ calculation in the above equation^15^. Based on the 457 CT data sets, an average D_W_ of 27.522 cm was calculated.

**Figure 1:**
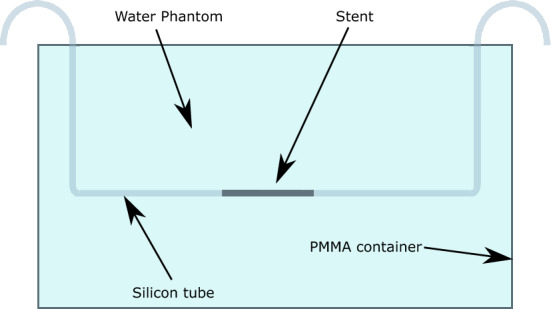
Phantom setup with a water equivalent diameter of Dw = 27 cm and the stent positioned in the isocenter. The phantom was built to reflect the water-equivalent diameter of an average adult patient based on actual CT scans of a random sample of 457 individuals (189 women, 268 men; median age 61; range 19–93 years, median BMI 27.23; range 17.2–58.77). BMI, Body mass index.

Based on this, a phantom was assembled as follows: a phantom made by polymethyl-methacylate (PMMA) with length of 36.0 cm, width 24.5 cm was filled with tap water. This design results to a D_W_ of 28.0 cm, shown in Fig. 1. For CT measurements, the different stents (see below) were inflated in appropriately sized silicone tubes using the pressure specified in the in vitro compliance table provided by the manufacturer. Subsequently, the silicon tubes were mounted into the isocenter of the phantom. We decided to use silicon tubes as vessel phantoms as the relatively low density of silicon did not affect image quality analysis in a trial setup proceeding the study.

In our study, a total of 12 different small vessel stents (peripheral as well as coronary) of different sizes (3–8 mm), designs (covered vs. non-covered) and vendors were investigated. A summary of the characteristics of all stents is given in Table 1. For all measurements, the tubes were filled with contrast medium (Ultravist 370, BAYER, Germany) using a dilution to obtain 300 HU at 120 kVp. To prevent contrast agent sedimentation, a pump (flintronic aqua pump) was integrated into the phantom setup to mimic a blood flow rate of 1 m/s.

**Table 1:** Investigated stents and their main characteristics with diameter [mm], length [mm], product name and specifications.

Stent No.	Diameter (mm)	Length (mm)	Product name	Manufacturer	Specifications
1	3.0	24.0	Promus ELITE MONORAIL	Boston Scientific	Everolimus-Eluting Platinum Chromium Coronary stent system
2	3.0	26.0	Synsiro	Biotronik	Sirolimus Eluting Coronary Stent System
3	3.0	15.0	PK Papyrus	Biotronik	Covered coronary stent system
4	3.5	26.0	PK Papyrus	Biotronik	Covered coronary stent
5	3.5	38.0	Resolute Onyx	Medtronic	Zotarolimus-Eluting coronary stent
6	4.0	26.0	Resolute Onyx	Medtronic	Zotarolimus-Eluting coronary stent
7	4.0	15.0	PK Papyrus	Biotronik	Covered coronary stent system
8	4.5	18.0	Resolute Onyx	Medtronic	Zotarolimus-Eluting coronary stent
9	5.0	27.0	Visi-Pro	Ev3	Balloon expandable peripheral stent system
10	6.2	30.0	ASSURAND Cobald	Medtronic	Over the wire iliac stent system
11	7.9	30.0	ASSURAND Cobald	Medtronic	Over the wire iliac stent system
12	8.0	100.0	Supera Vertias System	Supera Veritas	Peripheral vascular & biliary system

### CT Data acquisition and image reconstruction

#### PCD-CT

All PCD CT measures were performed on a NAEOTOM Alpha system (Siemens Healthcare GmbH, Forchheim, Germany) equipped with a cadmium telluride detector. Acquisition parameters for the multispectral cardiac CT protocol are summarized in Table 2.

**Table 2:** Acquisition parameters for the PCD-CT and EID-CT system.

Parameter	PCD-CT	EID-CT
Rotation time (s)	0.25	0.25
Collimation (mm)	144 × 0.40	136 × 0.60
Pixel size (mm)	0.40	0.60
Tube voltage (Kv)	120	120
Quality ref. mAs	74	400
Care keV IQ level	135	NA
Scan mode	sequence	sequence
Tube current (mAs)	68	94
Monoenergetic Energy Equivalent (keV)	60	–
Slice thickness (mm)	0.4	0.5
Increment (mm)	0.3	0.5
Matrix size	512 × 512	512 × 512
FoV (dual source) (cm)	50 & 36	50 & 35,5
Reconstruction type	QIR	ADMIRE
Reconstruction strength	3	3

PCD-CT, Photon-counting detector CT; EID-CT, Energy integrating detector CT.

From the acquired data, three series were reconstructed using the dedicated vascular kernel with increasing strength (soft (Bv40); intermedium (Bv48); high (Bv56)) were reconstructed. Kernel strength was initially selected based on a subjective consensus reading, where no subjective differences were found between Bv44 and Bv48 as well as between Bv56 and Bv60, respectively. Sharper reconstruction kernels beyond Bv60 were not assessed due to a substantial increase in image noise and reduced image quality. All images were reconstructed with a slice thickness of 0.4 mm and an increment of 0.3 mm. The Quantum Iterative Reconstruction (QIR) strength was 3. The QIR strength was selected to maintain the subjective image impression of the clinical standard EID-CT protocol.

#### EID-CT

All EID-CT scans were performed on a third generation dual-source CT system (SOMATOM Force; Siemens Healthcare GmbH, Forchheim, Germany) using the established clinical routine cardiac protocol serving as the reference standard. The EID-CT acquisition protocol parameters are summarized in Table 2.

The EID-CT series were reconstructed using the BV40 vascular kernel, a slice thickness of 0.5 mm and an increment 0.5 mm. The iterative reconstruction strength (ADMIRE; Siemens Healthineers; Forchheim, Germany) was set to 3.

### Image analysis

#### Qualitative image analysis

Image quality of the different reconstructions using the PCD and the EID CT-scanners was subjectively assessed by five radiologists (J.T.,J.W.,N.V.,M.D.,A.R.) for each stent separately, with 5–8 years of experience in cardiovascular imaging, independently in a random manner and blinded to the scanner and type of reconstruction. The criteria included (1) overall image quality, (2) sharpness, (3) subjective image noise, (4) blooming and (5) diagnostic confidence, where a 5-point Likert scale (1 = non-diagnostic, 5 = excellent) was implemented for scoring. All reading sessions were performed on a clinically approved workstation using the Picture Archiving and Communication System Deep Unity (Dedalus HealthCare, Bonn, Germany). Image data were provided to the readers in 0.4 mm reconstructions in axial and sagittal orientation.

#### Quantitative image analysis

**Figure 2:**
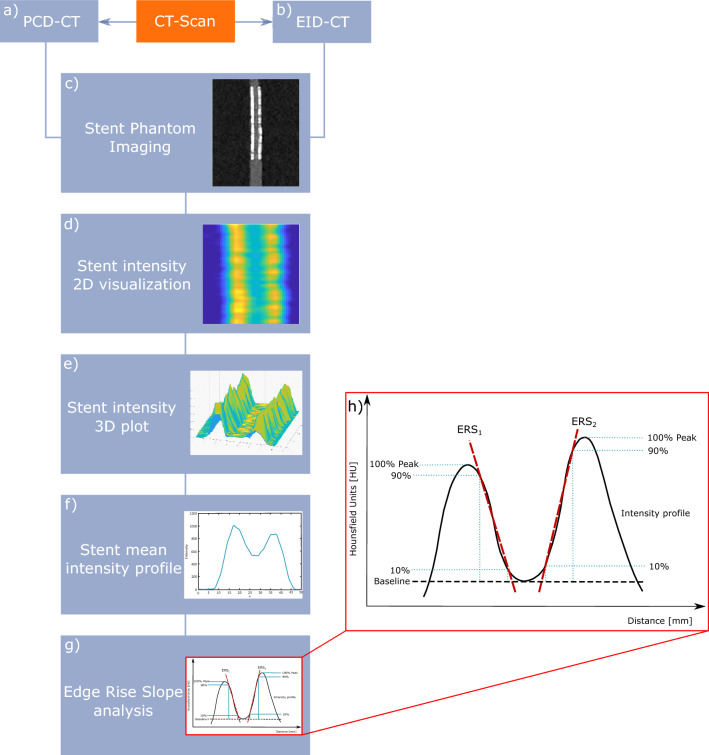
Overview of the quantitative image analysis pipeline using an in-house developed MATLAB script. All stents were scanned on (**a**) PCD-CT and (**b**) EID-CT. (**c**) CT DICOM dataset of every stent was reconstructed, (**d**) 2D intensity profiles were calculated for each stent, (**e**) and plotted as 3D intensity plot across the entire length of the stent, (**f**) mean intensity profile of the 3D intensity profile, (**g**) automatic estimation of the ERS of every stent and kernel, (**h**) mean intensity-profile curve of the stent with the edge rise slope a HU of 10% and 90% of the maximum CT attenuation are shown. PCD-CT, Photon-counting detector CT; EID-CT, Energy integrating detector CT; DICOM, Digital imaging and communications in medicine; ERS, Edge rise slope; HU, Hounsfield Units

To enable an objective quantification of the effect of the different kernels on the stent visualization an automatic algorithm (MATLAB software, MATLAB:2020b, The Mathwork Inc, Natick, Massachusetts) was developed to calculate the mean Edge Rise Slope (ERS) based on the attenuation of each stent struts (Fig. 2). The aim of this approach was to estimate differences in blooming/obscuration of the stent lumen considered as the most important feature for reliable stent assessment. Blooming was defined as blurring of interfaces and overestimation of stent size due to partial volume averaging.

Firstly, the sagittal cross-sectional image of each case through the axis of the phantom (axis of the stent) was reconstructed from the acquired data. Subsequently, the algorithm formed and averaged a large number, dependent on the stent size, over every voxel, the attenuation profiles perpendicular to the stent longitudinal axis were calculated and an average attenuation profile was then defined.

Thereafter, the width of the edge of the stent struts was measured, defined by the 10%-90% edge-rise distance (ERD) (Fig. 2b). Finally the ERS was calculated as follows:

2 $$ERS = \frac{HU90\% - HU10\% }{{ERD}}$$

The ERS was defined for both sides of the stent, and the mean value ERS_mean_ was calculated. In each data set, the minimum HU-values between the two maxima of the stent profile curves were measured. We decided to use the 10–90% interval based on the assumption that the edge of the signal rises most between these points and thus provides the most representative values of the gradient as a standardized method for the evaluation of the stents, independent of stent type and kernel.

In addition, the coefficient-of-variation (CV) was calculated for all stents and kernels, which is a commonly used measure to evaluate the homogeneity of the acquired signal as traditional quality measurements. To reduce measurement error, four individual ROIs of equal size 100 mm^2^ were positioned in the water area around the stent in a distance of 0.5 cm on axial reconstructions.

CV was calculated as follows:

3 $$CV = \frac{\sigma }{{\overline{HU} }}$$

where $\sigma$ is the mean standard deviation (SD) of HUs for ROI_1–4_ and $\overline{HU }$ the mean Hounsfield Unit in ROI_1–4_.

### Statistical analysis

Statistical analysis was performed using R (version 3.6.3; R Core Team, https://www.R-project.org). Due to the exploratory nature of this analysis, no formal power calculation was possible. Continuous variables are reported as mean ± SD or median and interquartile ranges (IQR) as appropriate. Categorical variables are presented as median and IQR. Qualitative reading scores were compared using Friedman´s ANOVA. For post-hoc pairwise comparisons the Wilcoxon rank sum test with continuity correction was conducted. For interobserver agreement, Fleiss’ κ was calculated and interpreted as follows: < 0.20 poor; 0.20–0.39 fair; 0.40–0.59 moderate; 0.6–0.79 substantial; > 0.80 perfect. Quantitative measures of the ERS analysis were compared using the repeated measure ANOVA. All p-values are two-sided and corrected for multiple comparisons using the Bonferroni method. The statistical significance level was set to 0.05.

## Results

### Image analysis

#### Qualitative image analysis

**Figure 3:**
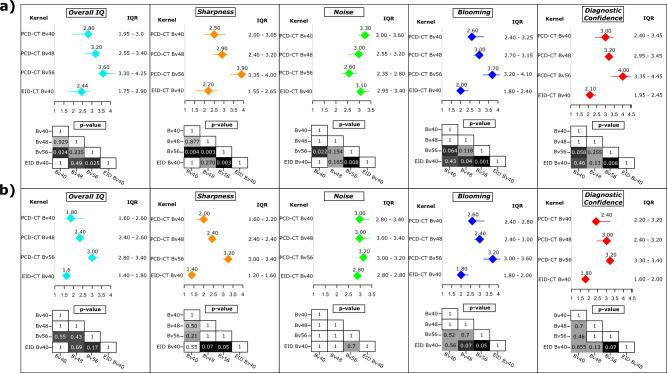
Result of the qualitative image analysis for (**a**) all stents and (**b**) stents limited to a diameter ≤ 3.5 mm. Results are presented as median and IQR. Results are shown for all readers. The *p*-values are calculated for the kernel comparison and presented as cross table for each image characteristic evaluation. All *p*-values are Bonferroni corrected for multiple comparisons. IQR, Interquartile ranges.

**Figure 4:**
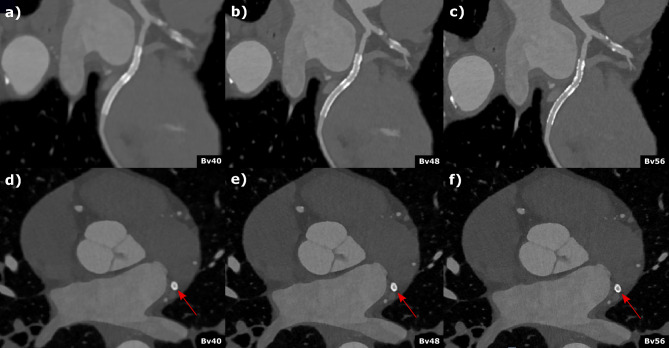
Coronary computed tomography angiography of a 62-year-old male patient with known coronary artery disease and stenting of the circumflex artery. Curved (**a**–**c**)) multiplanar reformations and axial (**d**–**f**)) reconstructed with different kernel strength (**a** and **d**) Bv40, (**b** and **c**) Bv48, (**c** and **f**) Bv56 depict the stent (2.5 mm diameter) in the circumflex artery. Stent lumen was best visible in the Br56 kernel reconstruction (**c** and **f**) and an in-stent restenosis could be reliably excluded. Scan was performed with 144 × 0.4 mm dual source, multi spectral high-pitch-flash-mode (3.2). Effective radiation dose was 1.07 mSv.

All reading sessions were completed by all five radiologists. A summary of the reading results is presented in Fig. 3. An example of image analysis for the different kernels is shown in Fig. 4.

Friedman ANOVA for overall image quality revealed significant differences between the different PCD-CT and EID-CT reconstructions (Friedman´s Q (df = 3) = 22.1; *p* < 0.001). Post hoc pairwise comparison showed the highest reading scores for PCD-CT Bv56 (3.6 [3.3–4.3]) followed by PCD-CT Bv48 and PCD-CT Bv40 (3.3 [2.8–3.5] and 2.8 [2.0–3.0]), respectively. The lowest ratings were found for the standard EID-CT Bv40 reconstruction (2.4 [1.8–2.9]). After Bonferroni correction to account for multiple testing, these differences were significant for PCD-CT Bv56 vs. PCD-CT Bv40 and EID-CT Bv40, respectively (*p* ≤ 0.04). A similar pattern was seen for sharpness and blooming. For image noise, Friedman ANOVA also revealed a statistically significant difference between the PCD-CT and EID-CT reconstructions (Friedman´s Q (df = 3) = 15.7; *p* = 0.001). In post hoc analyses, the most severe subjective image noise ratings were found for the PCD-CT Bv56 (2.6 [2.4–2.8]) and Bv48 kernel (3 [2.6–3.2]) followed by EID-CT Bv40 and PCD-CT Bv40 (3.2 [3.0–3.4] and 3.3 [3.0–3.6], respectively). In pairwise comparison, there was a significant difference between PCD-CT Bv56 vs. PCD-CT Bv40 and EID-CT Bv40, respectively (*p* ≤ 0.02) after Bonferroni correction. Significant results were also found for diagnostic confidence (Friedman´s Q (df = 3) = 24.1; *p* < 0.001) with the highest scores in post hoc testing for PCD-CT Bv56 (4 [3.6–4.5]). The ratings for PCD-CT Bv48, Bv40 and EIC-CT Bv40 were 3.2 [3.0–3.5], 3.0 [2.4–3.2] and 2.2 [2.0–2.6], respectively. In pairwise comparison, a statistically significant difference was found between PCD-CT Bv56 vs. PCD-CT Bv40 and EID-CT Bv40 after Bonferroni correction (*p* ≤ 0.2). Similar results were found in a subanalysis limited to stents ≤ 3.5 mm (Fig. 3). Inter-reader agreement across all readers and kernels was fair with a Fleiss kappa between 0.23 and 0.33. These results could already be proven in an initial scan of a patient with a 2.5 mm diameter stent, shown in Fig. 4.

#### Quantitative image analysis

**Figure 5:**
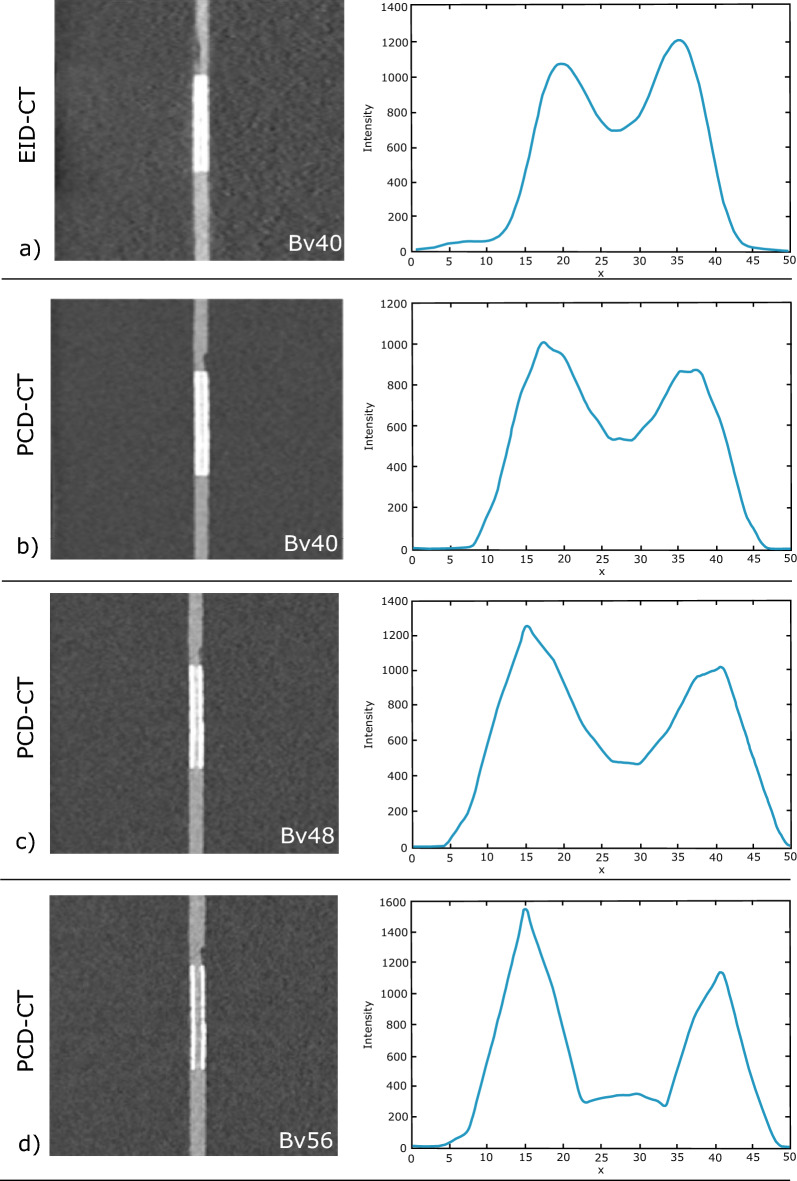
Image example of a 3 mm coronary artery stent (Everolimus-Eluting Platinum Chromium). Gray images depict the stent in EID-CT and three different kernel strengths of PCD-CT with the corresponding mean intensity profiles of the edge rise slope analysis. Subjective as well as objective analyses revealed higher image sharpness and reduced blooming with increasing kernel strength. PCD-CT, Photon-counting detector CT; EID-CT, Energy integrating detector CT.

An example of automatic calculation of attenuation profiles is shown in Fig. 5.

The summary of the ERS-based analysis for all stents is provided in Fig. 5, where a graded increase in ERS with increasing kernel strength is demonstrated. Repeated measures ANOVA revealed a significant effect of kernel strength on ERS (df_3_ = 32.5; *p* < 0.001). Post hoc pairwise comparison showed the significantly lowest mean ERS for PCD-CT Bv40 (ERS = 460.6 ± 179.34) followed by the clinical standard EID-CT Bv40 reconstruction (ERS = 456.2 ± 151.5) and PCD-CT Bv48 and Bv56, respectively (ERS = 770.8 ± 315.6) and (ERS = 1303.2 ± 414.4) (all *p* ≤ 0.05 corrected for multiple testing).

Results of CV analyses across all stent sizes are provided in Fig. 5 indicating a graded decrease in CV with increasing kernel strength in PCD-CT (Bv40 = − 3.73 ± 1.05, Bv48 = − 6.04 ± 2.67, Bv56 = − 5.11 ± 8.50 and a significantly higher in CV for the EID CT reconstruction (Bv40 = 10.49 ± 4.55) (all *p* ≤ 0.05 corrected for multiple testing).

**Figure 6:**
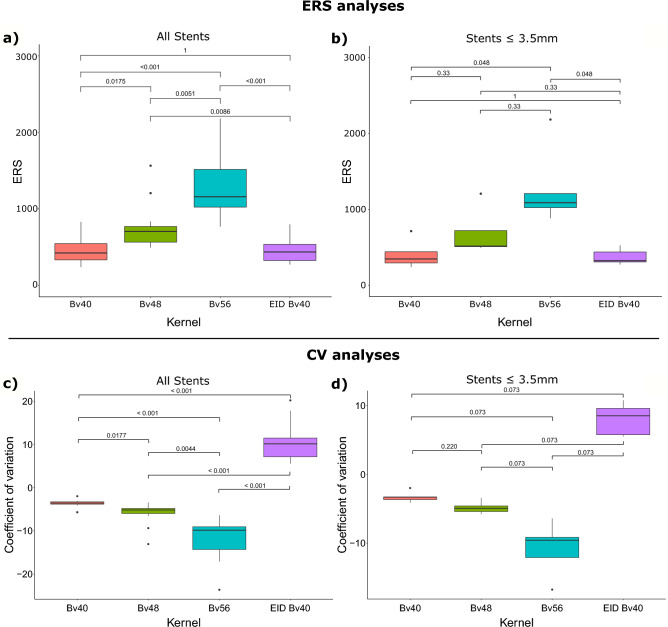
Results of the quantitative image analysis. Panel (**a**) and (**b**) show results of the edge rise slope analysis. Results of the coefficient-of-variation analysis are presented in panel (**c**) and (**d**). ERS, Edge rise slope; CV, Coefficient-of variation.

In a subanalysis limited to stents ≤ 3.5 mm similar results were observed for the ERS and CV-based analysis (Fig. 6). These results could already be proven in an initial scan of a patient with a 2.5 mm diameter stent, shown in Fig. 4.

## Discussion

We systematically investigated the value of PCD-CT on image quality and diagnostic confidence across a wide variety of small vessel stents. Our results indicate that PCD-CT with a sharp vascular kernel (Bv56) facilitates the most favorable image quality and diagnostic confidence with reduced artifacts and signal inhomogeneity when compared to other PCD-CT kernels and to a state-of-the-art third generation dual-source CT system.

These results are of clinical importance as recurrent symptoms in patients after PCI are common and in-stent restenosis is frequent^16^. A non-invasive reliable reevaluation method remains a key requirement in this context. In the latest guidelines for management of chronic coronary syndrome^17,18^, CCTA was implemented as a class I recommendation to diagnose or rule out chronic coronary syndrome in patients with low to intermediate pretest probability but it is not recommended when impaired image quality is expected^18^. This, however, is often observed after stent implantation and, thus, not routinely performed in these patients. A SCCT expert consensus recently published in 2021 recommended potential use of CCTA in patients with stable coronary artery disease after stent implantation if appropriate measures are taken to improve image quality (e.g., heart rate control, iterative image reconstruction and sharp reconstruction kernels)^9^. However, standard CT protocol recommendations do not currently exist. With the introduction of the first clinically approved photon-counting CT in late 2021, a new possibility to overcome these challenges might have become available^19^.

Our findings demonstrate that PCD-CT with dedicated vascular kernels provides high diagnostic image quality across a wide variety of stents and a significant reduction in blooming artifacts with increasing kernel strength. The most favorable image quality was found for an intermediate sharp kernel (Bv56) in both qualitative and quantitative analyses and was verified in initial patient scans. These results are in line with previous studies who report a significantly superior evaluation of in-stent lumen compared to conventional CT systems due to reduced blooming artifacts, image noise and higher spatial resolution^11,20,21^. Whether further improvement of the protocol proposed in this study can be achieved by utilizing the ultra-high resolution (UHR) mode with 0.2 mm pixel size as previously reported for a different reconstruction kernel^20,22^ needs to be evaluated in future studies.

A key feature of image analysis is the signal homogeneity of the acquired data^23^. Our CV analyses revealed that the overall image signal for all evaluated PCD-CT kernels was significantly more homogeneous compared to the EID-CT system regardless of the reconstruction kernel, which supports the observed results of the qualitative and edge rise slope analysis but may also allow for improved postprocessing in the future (e.g. monoenergetic reconstruction or advanced deep learning analysis) as the variance of the input data is reduced.

Our results are in line with previous studies^11,20,24^. For example, Verels et al.^25^ also reported an improved visualization of stents using photon counting CT compared to EID-CT in an ex vivo phantom setup similar to our findings. Decker et al.^26^ demonstrated better lumen visibility using sharp vascular kernels which was also reported by Elias Michael et al.^27^ who found improved image quality for in stent visualization using sharp reconstruction kernels combined with UHR acquisition mode.

The following limitations need to be considered. First, the EID-CT protocol was optimized for general cardiac imaging as a direct head-to-head comparison between the two scanners was not the primary focus of this study and stent evaluation is not a routine examination with established protocol recommendations. Second, we did not investigate stents with a diameter < 3 mm. Third, we only investigated the impact of different kernel strengths but did not explore the value of additional reconstruction settings (such as monoenergetic reconstructions (k-edge imaging) or QIR strength) to avoid confusion in data interpretation. Furthermore, indication-specific evaluations of stents such as calcium overlapping stents should be investigated in more detail. For accurate evaluation of in-stent restenosis, a dynamic cardiac phantom should be used to simulate and investigate the challenges caused by cardiac motion. Future studies are necessary to systematically assess these technical possibilities to further improve the proposed imaging protocol and compare to optimized standard approaches. In the current study, only three of potential five reconstruction kernels were investigated based on preliminary results proceeding the current study. Here we found no relevant difference between a Bv40 and Bv44 kernel as well as between a Bv56 and Bv60 kernel except of a significantly increased image noise. Therefore, we decided to provide results on a soft, medium and sharp kernel to demonstrate different use cases. Furthermore we did not evaluate data acquisition in UHR mode, due to the increase of radiation dose. This should be addressed in further studies.

In conclusion, we are the first study to demonstrate, by phantom setup adapted to real patient settings, that PCD-CT with a sharp vascular kernel (Bv56) provides the most favorable image quality for small vessel stent imaging and may serve as a starting point for further protocol optimization. Confirmatory studies translating our findings into clinical routine are needed to investigate whether PCD-CT has the potential to reduce the number of invasive coronary angiograms in the future.

## Data Availability

The data sets generated and/or analyzed in the current study are not publicly available, as this was agreed upon with the Institutional Review Board, but are available upon reasonable request from the corresponding author.

## Abbreviations

ANOVA: Analysis of variance
BMI: Body Mass Index
CCTA: Coronary computed tomography angiography
CV: Coefficient of variation
EID: Energy integrating detector
ERD: Edge rise distance
ERS: Edge rise slope
PCD-CT: Photon counting detector computed tomography
HU: Hounsfield units
IQR: Interquartile ranges
PCI: Percutaneous coronary intervention
ROI: Region of interest
QIR: Quantum iterative reconstruction
